# Enhanced Anomaly Detection in IoT Networks Using Deep Autoencoders with Feature Selection Techniques

**DOI:** 10.3390/s25103150

**Published:** 2025-05-16

**Authors:** Hamza Rhachi, Younes Balboul, Anas Bouayad

**Affiliations:** IASSE Laboratory, Sidi Mohamed Ben Abdellah University, Fez 30050, Morocco; younes.balboul@usmba.ac.ma (Y.B.); anas.bouayad@usmba.ac.ma (A.B.)

**Keywords:** internet of things, anomaly detection, dataset, deep autoencoder, data pre-processing, feature selection, classification

## Abstract

An enormous number of the Internet of Things (IoT) applications and their networks have significantly impacted people’s lives in diverse situations. With the increasing adoption of these applications in various sectors, ensuring reliability and security has become a critical concern. Moreover, the network that interconnected IoT devices uses advanced communications norms and technologies to capture and transmit data. Still, these networks are subject to various types of attacks that will lead to the loss of user data. Concurrently, the field of anomaly detection for the Internet of Things (IoT) is experiencing rapid expansion. This expansion requires a thorough analysis of application trends and existing gaps. Furthermore, it is critical in detecting interesting phenomena such as device damage and unknown events. However, this task is tough due to the unpredictable nature of anomalies and the complexity of the environment. This paper offers a technique that uses an autoencoder neural network to identify anomalous network communications in IoT networks. More specifically, we propose and implement a model that uses DAE (deep autoencoder) to detect and classify the network data, with an ANOVA F-Test for the feature selection. The proposed model is validated using the NSL-KDD dataset. Compared to some IoT-based anomaly detection models, the experimental results reveal that the suggested model is more efficient at enhancing the accuracy of detecting malicious data. The simulation results show that it works better, with an overall accuracy rate of 85% and 92% successively for the binary and multi-class classifications.

## 1. Introduction

The Internet of Things (IoT) is now a reality, featuring a vast array of sensors and nodes; it is utilized for several applications. From small to large networks, the primary objective is sending sensor data to base stations. Despite that, these data are vulnerable to various factors that affect the quality of the data collected or the network’s performance. IoT devices establish connections with other network devices and services through the network layer, which transfers data to the application for analytics, and smart services after receiving it from the perception layer [1]. This layer is essential for managing reliability, energy consumption, and security issues. IoT networks are mainly used to collect, analyze, report, and predict information that will be used for future purposes.

Incorporating and applying machine learning in the IoT space presents some significant challenges that require some consideration. A key advantage is the ability to manage complex relationships across multiple IoT domains. For example, it is used in healthcare to detect anomalies in patients or anomalies in medical data. It is also used in manufacturing to identify product defects and predict future failures. In the IT sector, it is applied to monitor the performance of computer systems and ensure smooth operations by identifying unusual trends in server logs or securing them from attacks.

Despite years of research into cyber-security, these communications become an important target for hackers due to their powerful economic effect and profound impact on our lives. This is why cyber-security has moved to the forefront of priorities in IoT infrastructure. In addition to significant measures to improve IoT network protection, various critical vulnerabilities in IoT networks also pose threats, with the most frequent cyber-attacks including DDoS (Distributed Denial of Service), Ransomware, and botnet attacks that aim to disrupt IoT networks and exploit the advantage of their features. Moreover, the scale of data produced by these devices is increasing rapidly and may include confidential and sensitive information.

In this scenario, the outlier detection issue is one of the major problems that demands extensive research and innovative solutions. The primary objective is to identify outliers and classify them as insignificant errors that should be ignored or critical events that must be taken to prevent performance degradation. There is an immediate requirement for detection methods that are very stable and effective due to the advent of new wireless network assaults. While firewall technology and universal wireless network authentication systems can meet users’ basic security protection requirements, their protective abilities are still limited. Anomaly detection techniques aim to identify abnormal behaviors that differ from expected patterns. These techniques typically involve machine learning algorithms to classify data as normal or anomalous [2]. Various factors, such as dataset selection, types of algorithms used for learning, feature selection, and evaluation methods play a crucial role in constructing models.

This article presents an approach of Deep Autoencoder for detecting cyber-attacks in IoT networks. The method focuses on learning normal and malicious data patterns to identify anomalous network behavior. The use of this method is particularly beneficial for IoT networks, where collecting labeled data can be challenging due to the presence of unknown cyber-attacks. DAEs are capable of modeling complex nonlinear relationships within the data, something that simpler methods cannot achieve, which is crucial for effective anomaly detection in such networks. Furthermore, DAEs excel at filtering noise in the data, allowing the model to focus on significant anomalies. They can also be optimized efficiently on resource-constrained IoT devices, with their architecture fine-tuned to balance accuracy and computational cost, making anomaly detection more practical in these environments. In addition, one of the main goals behind developing this DAE is to simplify the complexity by minimizing the number of operations. This reduction makes the detection more practical for IoT networks.

Our deep autoencoder approach offers several advantages over existing models. In addition to competitive performance in terms of anomaly detection, our model also stands out for its low resource consumption and reduced execution time. These properties make it a practical and efficient solution for situations where resources may be limited. Compared with other models that have exploited the same data, our method strikes a good balance between performance and resource requirements, making it particularly valuable in contexts where speed and efficiency are essential.

The suggested DAE has been tested and evaluated using the standard open-source NSL-KDD dataset. A significant challenge is addressing the issue of class imbalance in this dataset. This occurs when the target variable is unevenly distributed, with some classes being significantly underrepresented compared to others. In such cases, traditional techniques often have difficulty in learning from the minority class effectively because the majority class tends to dominate the learning process. This imbalance frequently causes models to favor the majority class, and not perform well in the minority class. The class imbalance problem is prevalent in anomaly detection.

Although the method employed in this research is based on a widely recognized approach, our innovation lies in its adaptation to the NSL-KDD dataset, combined with an improved feature selection and pre-processing strategy, significantly increasing classification performance compared to previous studies.

Details of this article are structured below.

- Section 2 presents state-of-the-art and the most recent research concerning IoT Anomaly detection.
- Section 3 describes and discusses the research methodology of the proposed model, a description of the dataset used, the feature selection technique based on the ANOVA F-test, and an overview of the model architecture.
- Section 4 is about the experimental results, analysis, and performance evaluation.
- Comparison with existing methods and suggestions for improving performance are outlined in Section 5.
- Section 6 presents the conclusion and discusses future works.

## 2. State of the Art

IoT networks, contrary to traditional networks, have particular vulnerabilities. Due to their diversity, the variety of protocols used (such as MQTT, CoAP), and their limited resources, IoT devices and networks are often more vulnerable to attack. These vulnerabilities include risks of insufficient security, notably due to a lack of frequent updates, DDoS attacks that take advantage of IoT botnets, and information leaks due to inappropriate or non-existent encryption. These factors underline the importance of having security solutions that are specifically designed for the IoT, different from the classic methods used in traditional networks. In this context, anomaly detection becomes an essential strategy for identifying malicious behavior in IoT networks.

Anomaly detection is an important aspect of securing IoT networks and has been widely studied using different machine learning and deep learning techniques. There are several categories of anomaly detection techniques used in IoT networks: Traditional methods like signature-based and rule-based systems (such as Snort) are good at detecting attack patterns but struggle with new or zero-day attacks. In addition, varying Machine Learning methods, such as SVM, decision trees, and random forests, are commonly used to detect anomalies in supervised and unsupervised applications. These methods are difficult to use with the complex and nonlinear nature of IoT data. Deep learning methods like convolutional neural networks (CNN), recurrent neural networks (RNN), and long short-term memory networks (LSTM) have demonstrated superior performance by automatically learning complex patterns. Autoencoders are particularly effective for unsupervised anomaly detection by identifying anomalies based on reconstruction errors. The autoencoder model captures nonlinear features and has difficulty being sensitive to noise during training. The other models are effective and simple for structured data; however, they have high computational costs.

Although IoT anomaly detection has improved, there are still challenges that remain. The most significant challenges are listed below:

- Data Imbalance: Models trained on imbalanced datasets may exhibit bias towards normal classes, resulting in lower detection rates for rare but important anomalies.
- Scalability: Effective models that can handle data with constrained computing resources are required to handle high-dimensional data in IoT networks.
- Application: Models trained for one application may not generalize well to another. For example, a smart home model may not be able to detect anomalies in industrial IoT systems because they have different data patterns.
- Execution Time: High execution time makes it hard to use these models in environments with limited resources or to react quickly to threats on time.

Building on this concept, anomaly detection is recognized as a powerful method for identifying attacks and malicious activities in IoT networks. Studies and research in this field have explored various tools and techniques across multiple domains, employing machine learning and deep learning methods.

In this literature review, the researchers studied and proposed several approaches using different algorithms, each with its strengths and weaknesses. Some researchers choose simple and fast approaches, while others prefer more complex techniques that offer better performance but require more resources. The analysis indicates that, although some studies achieve good results on different datasets, many have not been validated in a variety of contexts, limiting their applicability.

Table 1 provides a literature review of different techniques used for anomaly detection:

## 3. Methodology

### 3.1. Dataset Description

NSL-KDD is an updated version of the original KDD 99 dataset, providing a useful reference dataset for researchers to evaluate and compare different anomaly detection methods. It fixes the problems in the KDD 99 dataset by removing duplicate records in both the training and testing sets, which shows much better reduction rates. In addition, enhancing the representation of minority samples in the test set, this improvement facilitates a more accurate differentiation of various models for anomaly detection. The training set in the NSL-KDD dataset is called KDDTrain+, while the testing set is called KDDTest+. Each instance of this data structure has 42 attributes. Of these attributes, 41 represent the properties of the data structure, and 1 attribute indicates the attack type. The attacks replicated can be categorized into four types: DoS (Denial of Service), Probe, U2R (User to Root), and R2L (Remote to Local). Thus, for every entry, any of these 41 attributes can be designated to the normal type or the attack type of record. Moreover, the values for attributes can be nominal (Protocol_type, Service, and Flag), binary, or numerical.

In this work, categorization will utilize labels, whereby all values linked to the label attribute will be converted into either ‘attack_type’ or ‘normal’. A summary overview of the NSL-KDD dataset employed for training and evaluation of anomaly detection models is presented in Table 2. For our analysis, we utilized the training dataset, which was divided into 80% for the training set and 20% for the testing set.

#### 3.1.1. Data Preprocessing

Most machine learning methods are not suitable for real data. The data are often incomplete, posing a challenge to accurate analysis. To address this, data preprocessing techniques come to the rescue by enabling machine learning algorithms to effectively process data for the development of a model. In addition, these techniques typically enhance model accuracy, and not only cleanse the data from errors but also convert it to a preset format. The purpose of this part is to assess the most commonly used data transformation techniques for the data preprocessing stage. Nearly all datasets used for anomaly detection have missing values; these values can be classified as a phenomenon that often occurs in practice. Several techniques attempt to solve this problem. A commonly employed method to address this issue involves converting missing values into a numerical format. One fundamental approach is to use a fixed numeric value in place of the missing values (zero is frequently used). Our primary focuses were data encoding and feature scaling to pretreat our NSL-KDD dataset.

#### 3.1.2. Data Encoding

Data encoding plays a crucial role as a preliminary step in machine learning or deep learning; it describes the procedure for transforming textual or category data into numerical representation so that algorithms can use it as input. The rationale behind encoding is to facilitate the utilization of data by algorithms, which typically operate with numerical data rather than categorical or text data. Model performance might be affected by the encoding technique used; thus, it is critical to choose the best one for each situation. In our work, we used two different techniques: label encoder and one-hot encoder, depending on the nature of each feature to be encoded. Moreover, the label encoder scans for labels spanning from zero to n − 1 and assigns numerical values to these labels. In addition, we employ the one-hot-encoding strategy to transform categorical features into n-dimensional binary code vectors to improve the training efficiency of the model [11]. Each category is represented by a binary vector where ‘1.0’ signifies the presence of the category and ‘0.0’ denotes the absence of any other categories. Let us consider the feature “protocol_type” as an example, which has three unique properties: “tcp” “udp” and “icmp”, each one is encoded into a 3-dimensional binary vector: [1.0,0.0,0.0], [0.0,1.0,0.0], and [0.0,0.0,1.0] in that order, Table 3 illustrates the One-Hot Encoder for the “protocol_type” feature. Similarly, within the NSL-KDD dataset, we obtained three categorical features, namely “protocol_type”, “service”, and “flag” exist, with 3, 70, and 11 different attributes. (As shown in Table 3), the following table (Table 4) shows the main variables used to analyze anomalies in IoT networks. These variables are standard and widely recognized in the literature.

#### 3.1.3. Feature Scaling

Applying feature scaling is crucial to ensure that all features are close to a similar scale. Thus, each feature has a similar weight and facilitates simpler processing by the model. This enhances the model’s performance by detecting anomalies relative to their values rather than absolute size.

By utilizing the StandardScaler function, we transform the features to have a single scale, which is a practice advantageous for our model. This involves subtracting the mean and dividing by the standard deviation for each feature, applied to both the testing and training datasets, ensuring that the features result in a standard deviation of one and a mean of zero.

In mathematical forms, the StandardScaler () applied for each ***X*** feature can be described as $X\prime =\frac{X-\mu }{\sigma }$ where

X′ Represents the standardized value.

X Represents the original value.

μ Stands for mean values.

σ Signifies the standard deviation values.

**Figure 1:**
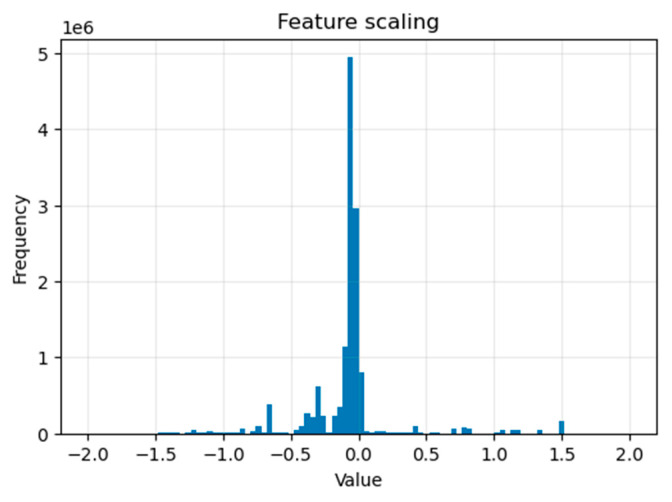
Feature scaling using the StandardScaler () function.

Figure 1 shows the result of applying the StandardScaler () function to our ‘x_train’ dataset (for the DoS attack) (we repeated for the other dataset ‘x_test’). It is frequently used on continuous numerical features to ensure that we have a mean of 0 and a deviation of 1.

### 3.2. ANOVA F-Test Based Feature Selection

Feature selection is an important step in the anomaly detection process, aiming to decrease figuring out applicable capabilities, cleansing data, and enhancing the accuracy of the detection model. This method involves choosing a subset of significant features from the entire collection of features, to eliminate any irrelevant features.

This technique analyzes how effectively each feature can separate attack classes. Features with high variance between classes and low variance within classes were favored because they facilitate a better distinction between normal and abnormal behavior, which improves model performance.

In our study, we implemented a univariate feature selection technique utilizing an analysis of variance (ANOVA F-test). This method allows us to assess the importance of each feature with the label by statistically evaluating the means of different groups using the F-test, i.e., each feature is selected and analyzed to find the most relevant feature score to the label. The intensity of the relationship between the feature and the label is calculated by examining each feature separately. The SelectPercentile method (sklearn.feature_selection) is used to determine features based on the percentile of the highest scores, comparing the data sample provided with a single specific value, which is considered outstanding. The value in this work is set at 0.1, where any values below this threshold are considered significant, and any value above it is considered non-significant.

When the scores are determined for each input feature, we can conclude that some features are more relevant than others, with much larger test statistic values. We have found that the ANOVA F-test provides better results for choosing the best NSL-KDD features for anomaly detection. The selected features for the four attack categories are detailed in Table 5.

According to this analysis, it is challenging to determine a single feature for each attack type since feature relevance varies based on the specific characteristics of the dataset and the classification assignment. For preference, we can readily identify the collective qualities present in all lists, as it is crucial to consider the overall significance of features within each class when making preferences.

After the data preprocessing in our application, we obtained 98 features. However, the common features chosen were 42 features; thus, we retained only the columns listed in Table 6 and dropped all other columns from the data frame.

In summary, the ANOVA F-test method has a major effect on performance by increasing accuracy, speeding up convergence, and reducing the number of features by reducing computational complexity, resulting in faster training and inference. These benefits make it a useful tool, particularly for high-dimensional datasets such as our NSL-KDD dataset.

### 3.3. Architecture of the Model

**Figure 2:**
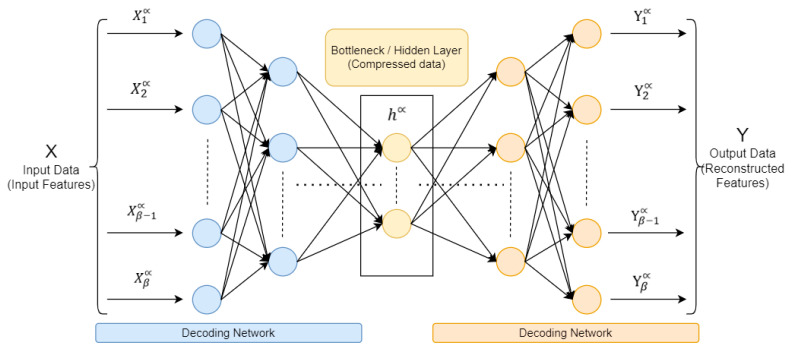
Deep autoencoder architecture.

A neural network is a set of interconnected processing nodes, often called “neurons”, which combine to convert different inputs into some desired action. An autoencoder (AE) is a feed-forward neural network reconstructing input data at the output layer. By reconstructing the input, the recording system attempts to understand the nature of the input data. In addition, the deep neural network is employed to replicate the input at the output layer, that is, the number of neurons in the output layer is equal to the number of neurons in the input layer [12]. A short time ago, the autoencoder models demonstrated improved performance in image and voice processing; these models are commonly employed in image anomaly detection, voice separation, and modeling/synthesis of language fields. The AE architecture, shown in Figure 2, comprises three main components: the encoder, the code (also known as the bottleneck), and the decoder. In addition, a deep autoencoder structure comprises multiple layers of neurons; depending on the particular task and dataset, there may be variations in the total number of layers and neurons in each layer.

In Figure 2, there are three primary parts in the autoencoder: encoder, bottleneck (code), and decoder. The encoder and decoder are fully interconnected, forming a feed-forward network, while the bottleneck serves as a distinct layer with it is possess size. It is characterized by the total number of nodes present in the intermediate layer.

The input features X, which stands for the input data and is represented by the formula: ***X*** = ($X_{1}^{\alpha }$, $X_{2}^{\alpha }$, $X_{3}^{\alpha }$, …, $X_{\beta -1}^{\alpha }$, $X_{\beta }^{\alpha }$), is transformed by the encoder into a hidden representation **h**, as seen in (1), which stands in for different levels of encoding and decoding. These layers create and generate an asymmetrical representation of the incoming data and reassemble it for the output layer. The bottleneck code is presented below, with σ (an activation function), **W** (a weight matrix), and **b** (a bias vector). Since, besides weights and biases, every neuron in a layer employs an activation function to determine its output by considering the weighted sum of its input data [13].

(1) $$h^{\alpha }=\sigma \;(W_{h}X^{\alpha }+b_{h})$$

An output data are Y = ($Y_{1}^{\alpha }$, $Y_{2}^{\alpha }$, $Y_{3}^{\alpha }$, …, $Y_{\beta -1}^{\alpha }$, $Y_{\beta }^{\alpha }$). This output, serving as a reconstructed copy of the input data, maintains the identical size as the input (as viewed in (2)).

(2) $$Y=\sigma \;(W_{Y}h^{\alpha }+b_{Y})$$

The performance of the deep autoencoder will be assessed using the reconstruction error, this value quantifies the accuracy of the reconstructed input by measuring the disparity between the output and the input. Therefore, the reconstruction error (RE) is calculated as the difference between the original input feature X and the reconstructed feature Y (3). Then, the reconstruction error RE is given by

(3) $$RE\;=\sum {\left(Y_{\beta }-X_{\beta }\right)}^{2}$$

Deep autoencoder parameters are tuned during the training step to minimize the RE for certain specific datasets. The reconstruction error will be minimal if the autoencoder is supplied data that is comparable to the data used in the training step after it has been trained. In contrast, if testing data deviates from training data, the reconstruction error will increase significantly.

#### The Proposed Approach

**Figure 3:**
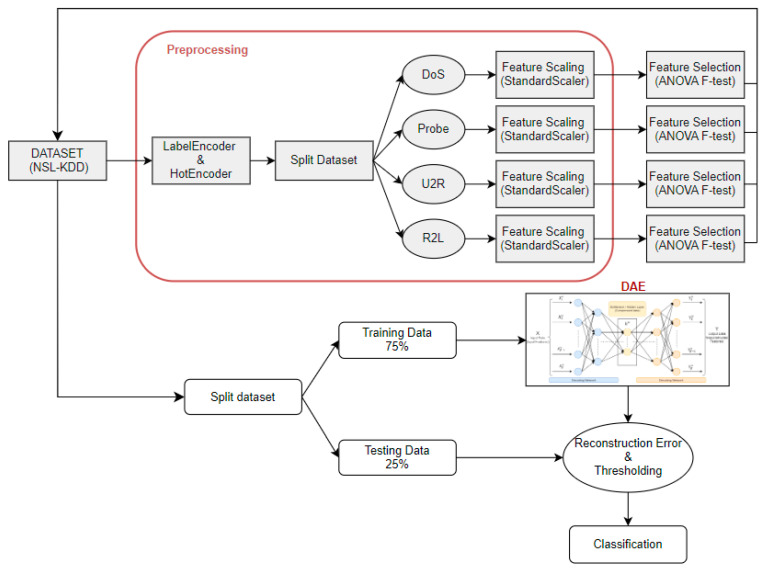
An overview of the proposed approach for anomaly detection.

The methodology of this research is presented in this section. Our approach involves employing a deep autoencoder to identify anomalies within IoT networks, and ANOVA F-test for better feature selection. Figure 3 shows the proposed structure.

This visual representation illustrates a comprehensive schematic of the architecture that we used in this paper using the NSL-KDD dataset (described in Section 3.1), a well-known dataset used for evaluating systems based on machine learning and deep learning. Specifically, focusing on the process of data preprocessing, it delineates multiple stages essential, progressing through encoding labels with “Label Encoder” and “Hot Encoder”, indicating the conversion of categorical data into a structure suitable for input into the model. In addition, we employed the StandardScaler technique for “Feature Scaling” and ANOVA F-test for “Feature Selection”. In addition, we have separated the dataset by class (DoS (denial of service), Probe, U2R (user-to-root), and R2L (remote-to-local)), selecting features for each class, which gives better results than applying them to the whole dataset.

Moreover, throughout the training process, we train our model using labeled data to calculate the reconstruction error and measure it between the original feature and the output feature. Therefore, we perform the classification using a threshold based on the best balance between sensitivity and specificity in the reconstruction error. This threshold value serves to classify traffic data into normal and anomalous categories [14]. That means any observations with a reconstruction error greater than the threshold are classified as anomalous, while those with a reconstruction error below the threshold are considered normal traffic data.

During the training process, our model was set with 20 training epochs, and a batch size of 64 to find a good compromise between training speed and stability of weight adjustments. Each deep autoencoder in our experiment has the following structural components:

The input layer has a size of 19, which corresponds to the total number of features.

The hidden layer consists of 30.

The output layer size is the same as the input layer size.

**Figure 4:**
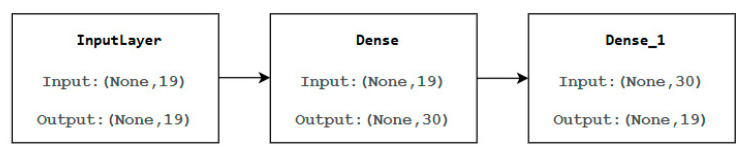
The diagram of our neural model.

The following diagram (Figure 4) is the diagram of our neural model, showcasing input and output processes. The diagram includes various components such as InputLayer, Dense, and Dense_1 layers with specific input and output dimensions.

**Figure 5:**
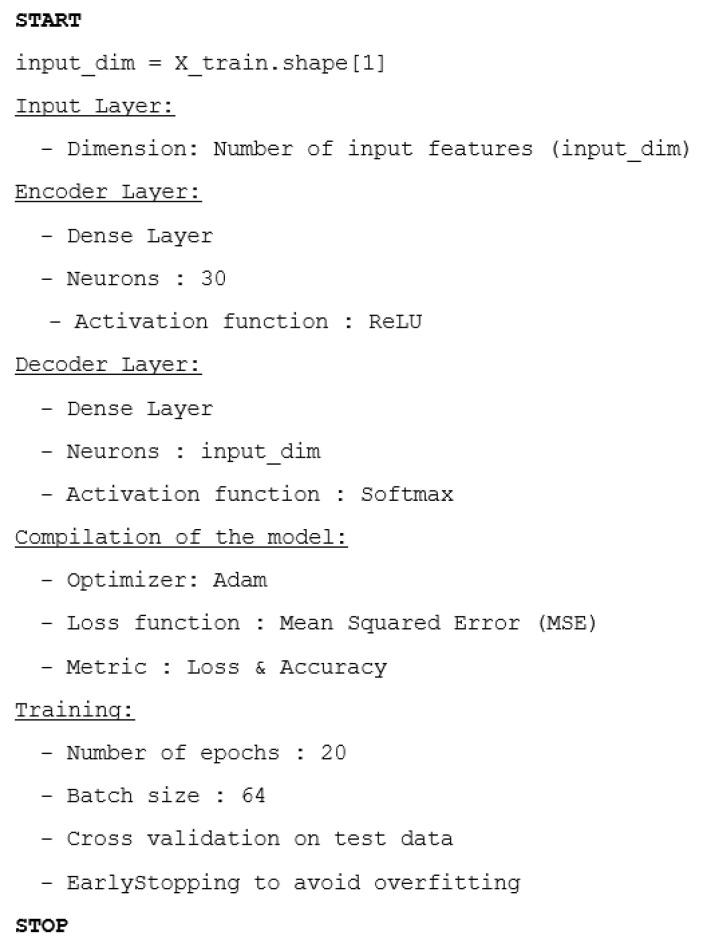
Detailed pseudocode of the model architecture.

The hyperparameters were selected based on several consecutive experiments. Following the notice of steady convergence without fluctuations, the training rate (Adam optimizer) was adjusted to the standard default value of 0.001. Activation function selection (ReLU) incorporates nonlinear behavior while ensuring good convergence. The number of neurons was progressively reduced in the hidden layers to optimize data compression, helping to identify anomalies. These decisions were confirmed by tests based on results obtained on the testing dataset. Moreover, to make it easier to understand the architecture and training process of the proposed deep autoencoder, we provide below (Figure 5) a pseudocode that summarizes the main steps used.

The early stopping technique was used to improve our training and avoid overfitting. This method monitors our model’s performance on the validation set by looking specifically at the validation loss (val_loss). If the validation loss does not show improvement within a predefined number of epochs (in our case: patience = 5) the training process stops. In addition, enabling the restore_best_weights parameter to true guarantees the model returns to the epoch with the lowest validation loss for the best performance. This method helps to reduce overfitting and improve the model’s capability to predict new data by avoiding excessive training.

## 4. Experimental Results

### 4.1. Experimental Setting

In this section, we look at the hardware and software programs used to increase the performance of our model. The experiment is carried out using a Windows 11 system with RAM 16 GB, an Intel Core i7 processor with 2.6 GHz, and The GPU model is NVIDIA GeForce^®^ RTX 2070. For environment and software, we have utilized Anaconda Jupyter Notebook (6.4.12) and Tensor Flow 2.4. 0. Moreover, Python (3.9.13) was used to write all the codes for conducting experiments, with specific emphasis on the Keras framework.

### 4.2. Performance Evaluation

We employ various performance metrics to evaluate the effectiveness of our proposed approach. We assess how well our suggested approach performs, utilizing the Area under the Curve (AUC). The following formulas were used to obtain the true positive rate (TPR) and false positive rate (FPR):

TPR = TP/TP + FN

FPR = FP/FP + TN

where

- TP (true positive): anomalies of instances that are accurately identified as anomalies.
- FN (false negative): anomalies of instances that are incorrectly classified as normal.
- TN (true negative): refers to normal instances that are accurately identified as normal.
- FP (false positive): refers to normal instances that are mistakenly classified as anomalies.

Without forgetting the metrics employed to evaluate the effectiveness of our proposed methodology using attribute values obtained from the training and testing processes of the NSL-KDD dataset. These values are outlined below:

Accuracy (AC):

(4) $$AC=\frac{TP+TN}{TP+TN+FP+FN}$$

Prediction (P):

(5) $$P=\frac{TP}{TP+FP}$$

Recall (R)

(6) $$R=\frac{TP}{TP+FN}$$

F-measure (F)

(7) $$F=\frac{2*P*R}{P+R}$$

### 4.3. Classification Results

The performance of our suggested approach in detecting IoT network anomalies was improved. Our efficiency architecture is based on the DAE model and ANOVA method. The NSL-KDD dataset is used to evaluate the model, since it has a large number of malicious network traffic from IoT networks, including the attack categories of DoS, Probe, U2R, and R2L. In addition, experiments have been performed aiming for improved performance in binary classification and multi-class classification.

#### 4.3.1. Binary Classification

Training and validation of binary classification took slightly less time than the multi-class classification. Moreover, the same tuning parameters used for binary classification were also applied to the multi-class classification. Adam optimization method yields superior performance when combined with early stopping and dynamic learning rates, which monitor the number of training epochs during the training step. Also, the dataset was categorized as either anomalous or typical network activity, and this classification achieved high precision, recall, F1 score, and accuracy, indicating minimal false positives (FP) and false negatives (FN) in predictions.

The evaluation report of binary classification is depicted in Table 7, presenting metrics for the two classes “0” and “1” (“0” is the normal class, and “1” is the anomaly class) including precision, recall, and F1-score. The report reveals that class “0” has a precision of 0.79, recall of 0.98, and F1-Score of 0.87. In contrast, class “1” demonstrates a precision of 0.97, recall of 0.70, and F1-Score of 0.81. Moreover, the overall accuracy of the classification is 0.85. The timing indicates that the model took 42.76 s to train on the dataset. Once trained, it requires 0.97 s to make predictions.

#### 4.3.2. Multi-Class Classification

The classification report in Table 8 summarizes the performance evaluation of our multi-class classification model. The dataset utilized to test our model included the following 5 classes: “normal”, “Dos”, “Probe”, “R2L”, and “U2R”. In addition to the overall accuracy and support for each class, the model’s evaluation metrics include precision, recall, and F1-score per class.

The model shows strong performance in detecting different types of network traffic across most categories. For DoS attacks, it achieves a perfect recall (1.00), meaning it identifies all actual DoS cases correctly, though with a precision of 0.81, indicating some false positives. In the detection Probe attack, the model is highly precise (1.00), but has a recall of 0.86, suggesting it misses some true instances. For R2L attacks, the model has a high precision (1.00) and recall (1.00), showing that it performs well in identifying this type of attack, achieving an F1-Score of 0.95, which indicates a strong balance between precision and recall. The model has perfect scores on U2R attacks in all metrics because this is based on a small sample size of 11 instances. For the normal category, the model has perfect precision, but misses some instances, as shown by the recall of 0.87. In summary, this classification demonstrates high performance, particularly in its precision across most classes, suggesting minimal false positives. Recall varies more for the “Normal” and “Probe” classes, where recall is lower than precision. The model has an overall accuracy of 92%. The timing metrics show that the model completed training in 44.03 s, representing the total time needed to learn from the dataset. After training, it takes only 4.51 s to make predictions, showing its efficiency in classifying new data rapidly. At this stage, the time required to train our deep autoencoder model is reasonable.

In light of the above, training a deep autoencoder model is a computationally intensive process that takes a significant amount of time and is heavily influenced by several factors. This is mainly affected by the network architecture’s complexity, the dataset’s size, and the hardware’s computational power.

### 4.4. Reconstruction Errors and ROC Curves

**Figure 6:**
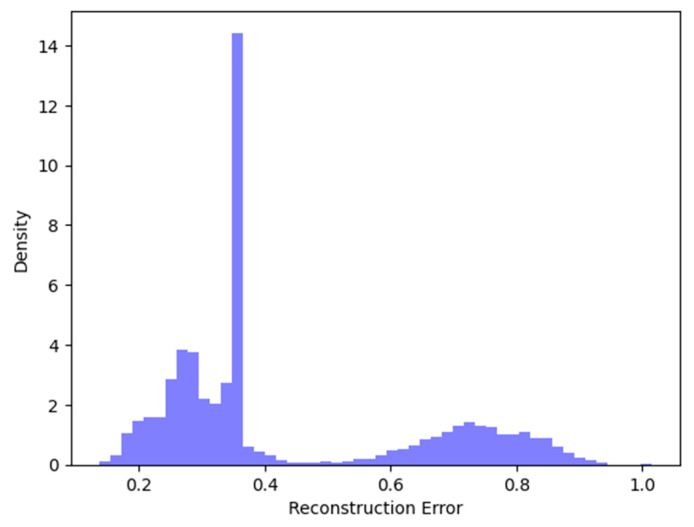
Distribution of reconstruction errors.

A study of the suggested model’s performance results is given in this section. Initially, optimal hyper-parameter values were chosen, resulting in the configuration of the final model with the ReLU activation function for the input layer and Softmax for the output layer. Our model was trained and set up utilizing Adam optimizer, with mean squared error as the loss function. After this, the mean square error (MSE) was determined between the reconstructed and the input data. Subsequently, an optimal threshold was estimated to isolate normal data and anomaly data. The reconstruction error distribution generated in our application is displayed in Figure 6. It is a well-organized diagram that visually represents a plot, with its main emphasis on the relationship between data density and reconstruction error. The values situated between 0.2 and 0.4 on the reconstruction error axis imply that most of the errors fall within this range; this could suggest that the majority of reconstructions were relatively accurate.

**Figure 7:**
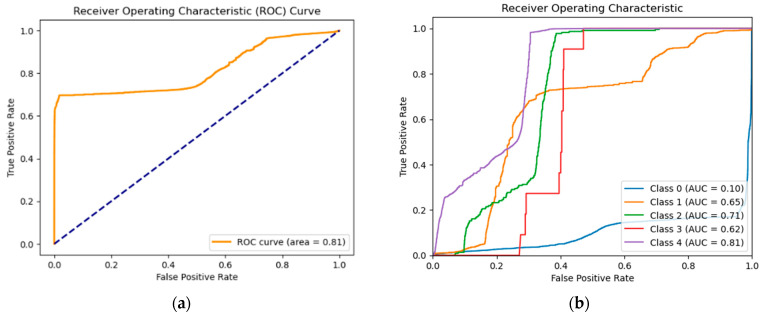
(**a**) ROC Curve for the binary classification. (**b**) ROC Curve for the multi-class classification.

In addition, to determine the construction error thresholds for both binary and multi-class classifications, we achieved the best possible balance between recall and precision. Consequently, the reconstruction error thresholds are shown in Table 9:

Additionally, we obtained the ROC curve (Receiver Operating Characteristic curve) by plotting TPR as opposed to FPR, which yields an area of 0.81 for the binary classification, as depicted in Figure 7. Also in Figure 7, there is a plot showing the Receiver Operating Characteristic (ROC) curve for different classes. Note that class 4 is the normal class; the other classes 0, 1, 2, and 3 are DoS, Probe, R2L, and U2R, respectively. Each line corresponds to a specific class and the area under each line represents the Area Under Curve (AUC) for that class, measuring the model’s performance and its ability to distinguish between positive and negative instances. By analyzing the AUC values for each class, we can identify the classes where the model performs well and those where it performs poorly. The ROC for class 0 is notably low (AUC = 0.10), indicating that the model cannot distinguish effectively between class 0 and other classes. This means the model has difficulty identifying class 0 instances and often misclassifies them as other classes.

Given that the score used to construct the ROC curves is based on global reconstruction errors and not on probabilities associated with each category. Some ROC curves may show poor performance for certain classes, even if the classic metrics (precision, recall, F1-Score) remain high.

**Figure 8:**
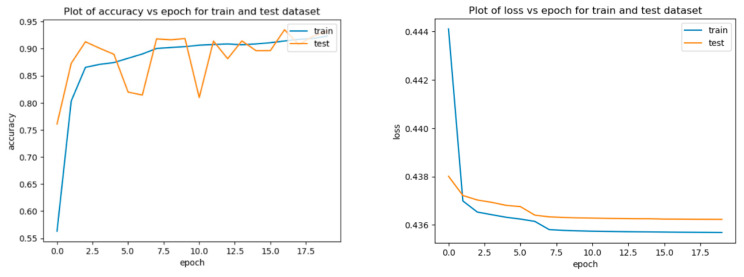
Variation curves of accuracy and loss rate with epochs for our model.

AUC Score provides a rapid comparison of several models and measures their performance in classification tasks. Generally, a model with a higher AUC performs better when tested on an identical dataset. Rather than visually evaluating a model using a ROC curve, the AUC condenses it into a solitary numerical measure, with a greater AUC suggesting better model performance. Table 10 shows the AUC values and interpretations:

The changes in loss rate and accuracy during the training of the Autoencoder are shown in Figure 8. During the first epoch, the Autoencoder’s loss rate begins to decrease for both the training and testing datasets, indicating that the model is learning and improving. This plot is a good indication that our model is performing well and is likely to be able to generalize well to new data.

However, both training and test accuracy have been increasing over time, showing that our model is successfully learning and improving. Overall, the graph demonstrates that the accuracy of the model decreases over a certain number of epochs. This is a common phenomenon in machine learning, and it is important to know when to stop training the model to avoid overfitting.

## 5. Comparison and Discussion

Anomaly detection is a topic of extensive research due to its critical importance in today’s IoT Cybersecurity world. Researchers have explored advanced machine learning and deep learning techniques for effective detection. Table 11 presents a comparative analysis of our proposed method with several previous approaches based on accuracy using the NSL-KDD dataset.

The table shows many techniques and their accuracies; our proposed method (DAE with ANOVA) has the highest accuracy of 92%, performing better than other methods. Samir Fenanir et al. [15] achieved the second-highest accuracy of 91% with deep autoencoder (DAE). Furthermore, the suggested method is more effective for detecting anomalies, since it performs better in accuracy and takes less time than the other DAE-based method, for training or decision.

To better highlight our contribution, researchers [20] used a deep autoencoder model (with 91% accuracy) on the NSL-KDD dataset and trained it only on normal data without applying specific feature selection techniques or advanced data processing. Although we also use the NSL-KDD dataset, we integrate feature selection techniques into our approach to remove attributes and improve model robustness. We use suitable scaling methods to enhance the quality of the autoencoder’s reconstruction. These improvements in data pre-processing explain the increase in accuracy we achieved.

Our approach achieves an excellent balance between performance, execution time, and simplicity. Nevertheless, it performs slightly inferior in accuracy to some more complicated models. The paper research [22] suggests a combination of DAE and SVM that achieves higher accuracy, but the model is more complex and takes 142.62 s in multi-classification. Due to the choice of the ANOVA F-test, our model is faster and more explainable. The study [23] proposed a method with a similar structure using a sparse autoencoder combined with logistic regression, reaching an accuracy of nearly 92.3%. However, their study did not specify the computational time required for the training and decision. In addition, their model had a false positive rate of 3.5%, which may affect its reliability in sensitive environments. Singh et al. [24] demonstrate improved accuracy (97.63%), but with a much longer processing time (taking up to 142.62 s for the DAE-SVM). Therefore, our method represents a suitable balance between accuracy, speed, and simplicity of deployment.

Anomaly detection is important for keeping data safe and secure, especially against malicious activities, and it needs more research by utilizing a variety of IoT data sources and offering comprehensible explanations for detected anomalies. Deep autoencoders are useful for detecting novel anomalies by identifying deviations in reconstruction errors and demonstrating robustness against noise, which is prevalent in IoT network data. Additionally, their scalability allows them to handle large datasets, ensuring real-time monitoring across vast IoT networks. However, they have certain limitations, including computational complexity, sensitivity to reconstruction error thresholds, and dependence on high quality. Their interpretability is also limited, making it difficult to understand the reasons behind flagged anomalies, and they may encounter difficulties in highly dynamic environments or when differentiating between various types of anomalies.

Although our approach shows encouraging results in the situations tested, certain limitations should be mentioned. For example, in rapidly evolving IoT environments, the method may require frequent adjustments to enhance its accuracy. Also, in zero-day attacks, detection can be even poorer. These limitations open several possibilities for improvement in the future.

## 6. Conclusions and Future Works

This paper focuses on improving anomaly detection methods, which are appropriate for the Internet of Things networks, with a particular emphasis on deep neural network utilization. The autoencoders in current research have garnered interest due to their capacity to handle large volumes of data and adjust to its dynamic nature. Their utilization has demonstrated encouraging detection and resolution of anomalies. This paper conducts an experimental analysis using the NSL-KDD dataset to evaluate the performance of the deep autoencoder. Moreover, to improve efficiency and reduce training process time, the dataset was pretreated in the first steps, and we used the ANOVA F-test method to select the significant features and use them for our model, we achieved the best accuracy with the implementation of our deep autoencoder model.

There are many opportunities for our future work to involve enhancing our model for deployment on embedded devices with limited resources by using techniques like quantization to minimize computational overhead while maintaining performance. And to extend this work, we also plan to examine advanced feature selection methods to improve the quality of input data, incorporate new data sources to enhance model efficiency, and explore other structures, such as ensemble learning.

## Figures and Tables

**Table 1:** Literature review.

Author(s) and Year	Technique(s) Used	Context
Doshi et al. [3]	SVM RF KN KNN DT NN	Employed virtual machines and Raspberry Pi to emulate IoT networks. They gathered network data from their system and subjected it to the test using five algorithms: support vector machine (SVM) accompanied by Linear Kernel, Random Forest, K-nearest Neighbors, Decision Tree, and Neural Network. The test set for all five algorithms had a greater accuracy.
Imtiaz Ullah et al. [4]	cGAN bcGAN FFN	Proposed an innovative framework for identifying Anomalies in IoT networks. Employing conditional Generative Adversarial Networks (cGANs) to address data imbalance and binary class Generative Adversarial Networks (bcGAN) for data enhancement. The model’s efficacy was evaluated using a feed-forward neural network (FFN) on datasets designed for network-based anomaly detection.
Imtiaz Ullah et al. [5]	LSTM BiLSTM GRU	Devised and put into action LSTM, BiLSTM, and GRU (Gated Recurrent Unit) models to establish a plan for analyzing anomalous activities in IoT networks. 7 datasets were employed to assess the effectiveness of these models compared to previous approaches. Compared to earlier methods, the multi-class and binary classifications demonstrated a high level of accuracy.
M.Hassan et al. [6]	LR SVM RF DT ANN	The authors compared various machine learning models to predict attacks and anomalies in IoT systems effectively. The machine learning algorithms utilized encompass Logistic Regression (LR), Support Vector Machine (SVM), Random Forest (RF), Decision Tree (DT), and Artificial Neural Network (ANN). The system’s testing accuracy for decision trees, random forests, and artificial neural networks reached 99.4%. Despite this shared accuracy, additional metrics indicate that Random Forest outperforms the other techniques.
Nilesh Kumar Sahu et al. [7]	ANN LR	Two machine-learning classifications are employed, and they present a comparison of their performances. The classification algorithms utilized include artificial neural network (ANN) and Logistic Regression (LR).
Zeeshan Ahmad et al. [8]	DNN CNN RNN GRU LSTM	Proposed deep neural networks (DNNs) for IoT networks using the IoT-Botnet2020 dataset. A comparative examination was performed on diverse deep learning models, including Convolutional Neural Network (CNN), and Recurrent Neural Network (RNN), along with their distinct variants like Gated Recurrent Unit (GRU) and Long Short-term Memory (LSTM).
Nicolas-Alin Stoian [9]	SVM NB RF ADA MLP	Concentrates on the security aspects of IoT networks, Examining the efficacy of machine learning algorithms in identifying anomalous data in IoT networks. Several algorithms, including Support Vector Machine (SVM), Naïve Bayes (NB), Random Forest (RF), AdaBoost (ADA), and Multi-Layer Perceptron (MLP), a variant of the Artificial Neural Network class, have been used employing various parameters and methodologies for comparison in a variety scenario. For the test, he used the IoT-23 dataset.
D.Thamaraiselvi et al. [10]	NB RF DT SVM	Focus on the IoT network features security, examining and investigating the usefulness and effectiveness of machine learning algorithms by identifying and detecting anomalies, within such network data. The study employs the following machine learning algorithms: Naive Bayes (NB), Random Forest (RF), Decision Tree (DT), and Support Vector Machine (SVM). Which have proven effective in comparable scenarios, and conduct a comparative analysis using various parameters and methodologies. Among these, the Random Forest algorithm attained the best results with an accuracy of 99.5%.

**Table 2:** Records per classes of the NSL-KDD dataset.

Category	Training Samples		Testing Samples	
Normal	67,343		9710	
DoS	45,927	58,360	7460	12,833
Probe	11,656		2421	
R2L	995		2885	
U2R	52		67	
Total	125,973		22,543	

**Table 3:** Encoding the Protocol_Type feature using the one-hot encoder.

	Protocol_Type_Icmp	Protocol_Type_Tcp	Protocol_Type_Udp
**0**	0.0	1.0	0.0
**1**	0.0	0.0	1.0
**2**	0.0	1.0	0.0
**3**	0.0	1.0	0.0
**4**	0.0	1.0	0.0

**Table 4:** Categorical features with different attributes.

Protocol_Type	Service	Flag
[‘protocol_type_icmp’, ‘protocol_type_tcp’, ‘protocol_type_udp’]	[‘service_IRC’, ‘service_X11’, ‘service_Z39_50’, ‘service_aol’, ‘service_auth’, ‘service_bgp’, ‘service_courier’, ‘service_csnet_ns’, ‘service_ctf’, ‘service_daytime’, ‘service_discard’, ‘service_domain’, ‘service_domain_u’, ‘service_echo’, ‘service_eco_i’, ‘service_ecr_i’, ‘service_efs’, ‘service_exec’, ‘service_finger’, ‘service_ftp’, ‘service_ftp_data’, ‘service_gopher’, ‘service_harvest’, ‘service_hostnames’, ‘service_http’, ‘service_http_2784’, ‘service_http_443’, ‘service_http_8001’, ‘service_imap4’, ‘service_iso_tsap’, ‘service_klogin’, ‘service_kshell’, ‘service_ldap’, ‘service_link’, ‘service_login’, ‘service_mtp’, ‘service_name’, ‘service_netbios_dgm’, ‘service_netbios_ns’, ‘service_netbios_ssn’, ‘service_netstat’, ‘service_nnsp’, ‘service_nntp’, ‘service_ntp_u’, ‘service_other’, ‘service_pm_dump’, ‘service_pop_2’, ‘service_pop_3’, ‘service_printer’, ‘service_private’, ‘service_red_i’, ‘service_remote_job’, ‘service_rje’, ‘service_shell’, ‘service_smtp’, ‘service_sql_net’, ‘service_ssh’, ‘service_sunrpc’, ‘service_supdup’, ‘service_systat’, ‘service_telnet’, ‘service_tftp_u’, ‘service_tim_i’, ‘service_time’, ‘service_urh_i’, ‘service_urp_i’, ‘service_uucp’, ‘service_uucp_path’, ‘service_vmnet’, ‘service_whois’]	[‘flag_OTH’, ‘flag_REJ’, ‘flag_RSTO’, ‘flag_RSTOS0’, ‘flag_RSTR’, ‘flag_S0’, ‘flag_S1’, ‘flag_S2’, ‘flag_S3’, ‘flag_SF’, ‘flag_SH’]

**Table 5:** Features chosen for the four attack categories.

Category of Attack	Features Selected
DoS	[‘logged_in’, ‘count’, ‘serror_rate’, ‘srv_serror_rate’, ‘same_srv_rate’, ‘dst_host_count’, ‘dst_host_srv_count’, ‘dst_host_same_srv_rate’, ‘dst_host_serror_rate’, ‘dst_host_srv_serror_rate’, ‘service_http’, ‘flag_S0’, ‘flag_SF’]
Probe	[‘logged_in’, ‘rerror_rate’, ‘srv_rerror_rate’, ‘dst_host_srv_count’, ‘dst_host_diff_srv_rate’, ‘dst_host_same_src_port_rate’, ‘dst_host_srv_diff_host_rate’, ‘dst_host_rerror_rate’, ‘dst_host_srv_rerror_rate’, ‘protocol_type_icmp’, ‘service_eco_i’, ‘service_private’, ‘flag_SF’]
U2R	[‘urgent’, ‘hot’, ‘root_shell’, ‘num_file_creations’, ‘num_shells’, ‘srv_diff_host_rate’, ‘dst_host_count’, ‘dst_host_srv_count’, ‘dst_host_same_src_port_rate’, ‘dst_host_srv_diff_host_rate’, ‘service_ftp_data’, ‘service_http’, ‘service_telnet’]
R2L	[‘src_bytes’, ‘dst_bytes’, ‘hot’, ‘num_failed_logins’, ‘is_guest_login’, ‘dst_host_srv_count’, ‘dst_host_same_src_port_rate’,vdst_host_srv_diff_host_rate’, ‘service_ftp’, ‘service_ftp_data’, ‘service_http’, ‘service_imap4’, ‘flag_RSTO’]

**Table 6:** Features selected for our application.

Features chosen	[‘logged_in’, ‘count’, ‘serror_rate’, ‘srv_serror_rate’, ‘same_srv_rate’, ‘dst_host_count’, ‘dst_host_srv_count’, ‘dst_host_same_srv_rate’, ‘dst_host_serror_rate’, ‘dst_host_srv_serror_rate’, ‘service_http’, ‘flag_S0’, ‘flag_SF’, ‘rerror_rate’, ‘srv_rerror_rate’, ‘dst_host_diff_srv_rate’, ‘dst_host_same_src_port_rate’, ‘dst_host_srv_diff_host_rate’, ‘dst_host_rerror_rate’, ‘dst_host_srv_rerror_rate’, ‘Protocol_type_icmp’, ‘service_eco_i’, ‘service_private’, ‘src_bytes’, ‘dst_bytes’, ‘hot’, ‘num_failed_logins’, ‘is_guest_login’, ‘service_ftp’, ‘service_ftp_data’, ‘service_imap4’, ‘flag_RSTO’, ‘urgent’, ‘root_shell’, ‘num_file_creations’, ‘num_shells’, ‘srv_diff_host_rate’, ‘service_telnet’, ‘intrusion’, ‘abnormal’, ‘normal’, ‘label’]

**Table 7:** Binary classification results.

	Precision	Recall	F1-Score	Support
**“0”**	0.79	0.98	0.87	13,422
**“1”**	0.97	0.70	0.81	11,773
**Accuracy**			0.85	25,195
**Macro avg**	0.88	0.84	0.84	25,195
**Weighted avg**	0.87	0.85	0.84	25,195
			Training Time	42.76 s
			Decision Time	0.97 s

**Table 8:** Multi-class classification results.

	Precision	Recall	F1-Score	Support
**DoS**	0.81	1.00	0.90	9181
**Probe**	1.00	0.86	0.92	2357
**R2L**	1.00	0.91	0.95	224
**U2R**	1.00	1.00	1.00	11
**Normal**	1.00	0.87	0.93	13,422
**Accuracy**			0.92	25,195
**Macro Avg**	0.96	0.93	0.94	25,195
**Weighted Avg**	0.93	0.92	0.92	25,195
			Training Time	44.03 s
			Decision Time	4.51 s

**Table 9:** Identification of thresholds for our deep autoencoder model.

**Classification**	**Multi-class**		**Binary**
	**Class**	**Threshold**	**Threshold**
	**DoS ‘0’**	**0.8358**	**0.3625**
	**Probe ‘1’**	**0.5336**	
	**R2L ‘2’**	**0.3350**	
	**U2R ‘3’**	**0.3592**	
	**Normal ‘4’**	**0.3611**	

**Table 10:** AUC interpretations.

AUC	Interpretation
AUC = 0.5	This means the performance of the model is equal to irregular estimation, On the ROC plot, it is represented by the diagonal line with vertices (0:0) and (1:1).
0.5 < AUC < 1	This shows that the model surpasses the irregular estimation, providing more effectiveness and can distinguish between negative and positive values, especially when the value is near to 1.
AUC = 1	It is a theoretical value that is rarely realized in practice, indicating that the model can perfectly distinguish between negative and positive values.

**Table 11:** Comparative analysis of our proposed method with several previous approaches.

Ref	Authors	Techniques	Datasets	Accuracy
[15]	Wen Xu et al.	5-Layer Autoencoder (AE)	NSL-KDD	90.91%
[16]	Mashuqur et al.	Light GBM (Gradient Boosting Machine)—K-Means	NSL-KDD	90.41%
[17]	Ieracitano et al.	Autoencoder (AE)	NSL-KDD	84.21%
[17]	Ieracitano, et al.	Long-Short-Term-Memory (LSTM)	NSL-KDD	82.04%
[17]	Ieracitano et al.	MultiLayer Perceptron (MLP)	NSL-KDD	81.65%
[18]	Arrun Sivasubramanian et al.	CAAE-DNN(Conditional Autoencoder using Deep Neural Networks)	NSL-KDD	79.18%
[19]	Arindam Sarkar et al.	Multi-Layer Perceptrons (MLPs)	NSL-KDD	87.63%
[20]	Samir Fenanir et al.	Deep Autoencoder (DAE)	NSL-KDD	91%
[21]	Shuokang Huang et al.	Imbalanced Generative Adversarial Network (IGAN)	NSL-KDD	84.45%
[22]	Khan and Mailewa	DAE + SVM	NSL-KDD	98.5%
[23]	Gurung et al.	Sparse Autoencoder (SAE) + Régression Logistique (RL)	NSL-KDD	92.3%
[24]	Singh et al.	Stacked Autoencoder/principal component analysis	NSL-KDD	95.71% (5 class)
	Our proposed	Deep Autoencoder with analysis of variance (DAE + ANOVA)	NSL-KDD	92%

## Data Availability

Data sharing is not applicable to this article.
